# Immunoprofiling of Equine Plasma against *Deinagkistrodon acutus* in Taiwan: Key to Understanding Differential Neutralization Potency in Immunized Horses

**DOI:** 10.3390/tropicalmed8010051

**Published:** 2023-01-09

**Authors:** Cho-Ju Wu, Geng-Wang Liaw, Chun-Kuei Chen, Chun-Hsiang Ouyang, Yi-Xiu Yang, Li-Chieh Chu, Yung-Chin Hsiao, Chien-Hsin Liu, Wen-Chin Hsieh, Cyong-Yi Wang, Yu-Syuan Liou, Chien-Chun Liu, Cheng-Hsien Hsieh

**Affiliations:** 1 Department of Emergency Medicine, Chang Gung Memorial Hospital, Linkou, Taoyuan 33305, Taiwan; 2 Department of Emergency Medicine, Yeezen General Hospital, Taoyuan 32645, Taiwan; 3 Master Program of Health Policy and Business Administration, College of Technology Management, National Tsing Hua University, Hsinchu 30013, Taiwan; 4 College of Medicine, Chang Gung University, Taoyuan 33302, Taiwan; 5 Department of Trauma and Emergency Surgery, Chang Gung Memorial Hospital, Linkou, Taoyuan 33305, Taiwan; 6 Molecular Medicine Research Center, Chang Gung University, Taoyuan 33302, Taiwan; 7 Department of Otolaryngology Head and Neck Surgery, Chang Gung Memorial Hospital, Linkou, Taoyuan 33305, Taiwan; 8 Graduate Institute of Biomedical Sciences, College of Medicine, Chang Gung University, Taoyuan 33302, Taiwan; 9 Center for Diagnostics and Vaccine Development, Centers for Disease Control, Ministry of Health and Welfare, Taipei 11561, Taiwan; 10 Department of Emergency Medicine, En Chu Kong Hospital, New Taipei City 23741, Taiwan; 11 Graduate Institute of Clinical Medicine, College of Medicine, Taipei Medical University, Taipei 11042, Taiwan

**Keywords:** antivenom, *Deinagkistrodon acutus*, neutralization potency, horse plasma, immunoprofiling, acutolysin A

## Abstract

Snakebite envenoming is a public health issue linked to high mortality and morbidity rates worldwide. Although antivenom has been the mainstay treatment for envenomed victims receiving medical care, the diverse therapeutic efficacy of the produced antivenom is a major limitation. *Deinagkistrodon acutus* is a venomous snake that poses significant concern of risks to human life in Taiwan, and successful production of antivenom against *D. acutus* envenoming remains a considerable challenge. Among groups of horses subjected to immunization schedules, few or none subsequently meet the quality required for further scale-up harvesting. The determinants underlying the variable immune responses of horses to *D. acutus* venom are currently unknown. In this study, we assessed the immunoprofiles of high-potency and low-potency horse plasma against *D. acutus* venom and explored the conspicuous differences between these two groups. Based on the results of liquid chromatography with tandem mass spectrometry (LC-MS/MS), acutolysin A was identified as the major component of venom proteins that immunoreacted differentially with the two plasma samples. Our findings indicate underlying differences in antivenoms with variable neutralization efficacies, and may provide valuable insights for improvement of antivenom production in the future.

## 1. Introduction

Snakebite is a critical medical issue in tropical and subtropical regions, as snake habitats frequently overlap with human settlements [1]. Every year, at least 81,000 people die from snake envenomation and more than 5,400,000 people are exposed to snakebite injury in subtropical regions worldwide [2,3]. Taiwan, a country located in east Asia, has made a considerable effort to combat the effects of snakebite [4]. *Deinagkistrodon acutus*, belonging to *Crotalinae* family, is one of the major snake species contributing to fatalities in Taiwan [5]. This venomous snake is also commonly known as the hundred-pace pit viper or five-pace snake, and its bite can lead to severe rapid-onset coagulopathy and thrombocytopenia in *D. acutus* envenomed victims. The symptoms of *D. acutus* envenomation are caused by two main components, metalloproteases and serine proteases, present in the venom of hundred-pace pit viper [6,7,8].

Serotherapy has so far been the mainstay treatment for envenomated victims, with documented efficacy in reducing the risk of mortality [9]. Antivenoms or so-called antitoxins include purified antibodies against snake venoms, extracted from vaccinated animals, especially horses and sheep [10]. These antibodies can neutralize the components of venom by inhibiting their toxic domains and in turn rapidly reduce the occurrence of envenoming symptoms [11]. While antivenom is currently the gold standard treatment for snakebite envenomation, it is associated with a number of limitations [11]. For instance, adverse reactions such as fever, chills, and nausea in patients following administration of antivenom are a matter of concern [12,13,14]. These reactions occur mainly due to the foreign nature of the immunoglobulins [15] that trigger the serum sickness [16]. Furthermore, antivenom is very expensive and the manufacturing procedure is time-consuming [17]. Consequently, many patients in rural countries can neither access nor afford this life-saving therapy [18,19,20], and several places including areas of south Asia and other developing countries lack effective antivenom supplements [21,22]. Most importantly, the efficacy of antivenoms can vary between batches. Even when animals used for the production of these antidotes belong to the same species and have been hyperimmunized with snake venom under identical conditions [23], in some cases antibodies are unable to bind to specific toxins in snake venom, usually leading to failure of antivenom against human envenomation [24]. An investigation into *Bothrops jararaca* antivenom in Brazil revealed the problem of divergent neutralizing ability among products. Antibodies from 26 different hyperimmunized horses showed variable efficacies and more than half the antivenoms were ineffective in neutralizing the injected venom in a mouse model [25]. The discovery that antivenom with adequate efficacy can only be produced from a few specific individuals poses a major challenge in antivenom production, and the reasons for these variations in affinity and ways to improve therapeutic efficiency are under widespread discussion [26,27,28,29].

Taiwan Centers for Disease Control (CDC) is the only institute that manufactures antivenom against *D. acutus* on this geographically isolated island [30]. Since 1960, the institute has provided clinics and hospitals throughout Taiwan with horse-derived antivenom, designated *D. acutus* Monovalent Antivenom (DaMAV) [31].

To produce DaMAV, snake venom is initially collected from caged hundred-pace pit vipers and then lyophilized. Horses bred by the CDC are repeatedly immunized with the purified venom protein and the neutralization potency of horse plasma is monitored using in vivo rodent assay. When the detected potency reaches the neutralization criteria, large-scale harvesting for antivenom generation is initiated. Although the procedure appears simple and practical, a serious obstacle remains unresolved. When several horses are immunized through multiple cycles, only a few or none produce antibodies that meet the quality for large-scale harvesting. This leaves the productivity of the current *D. acutus* antivenom strategy below expectations. Additionally, screening for effective equine sources before antivenom production leads to considerable financial costs and time expenditure. Elucidation of the factors underlying this variation in the immune responses of different horse-plasma samples toward venom proteins should represent a turning point in improving the treatment of *D. acutus* envenomation in Taiwan. To address this issue, we explored in the present study the immunoprofiles of hyperimmunized equine plasma against *D. acutus* venom and identified the differences in proteins between the high-potency and low-potency groups. Our data provide valuable insights into the underlying causes of dissimilar immune responses of horses to snake venom.

## 2. Materials and Methods

### 2.1. Snake Venom and Hyperimmunized Horse Plasma

*Deinagkistrodon acutus* venom and the hyperimmunized equine plasma samples were provided by the Centers of Disease and Control, Taipei, Taiwan. Crude venom was collected from 10 caged individuals, and mixed. Five naïve horses were immunized with 10 mg of whole venom every 14 days for eight cycles in schedule. The lyophilized venom was stored at −20 °C, and the plasma samples were stored at −80 °C before use.

### 2.2. Animals

Littermate ICR (CD1) mice (3 weeks old with a weight of 15–18 g) were used for the evaluation of the neutralization potency of hyperimmunized equine plasma. Mice were housed under a 12:12 h light–dark cycle and kept in a pathogen-free environment at a temperature of 22 °C and humidity level of 60–70%. Animals had *ad libitum* access to a standard rodent chow diet.

### 2.3. Animal Ethics Statement

Experiments involving animals and injection with various venoms were reviewed and approved by the Institutional Animal Care and Use Committee of Chang Gung University (Permit Number: CGU110-129). The protocol of the mouse study was based on the legal guidelines prescribed by the Taiwan animal protection act and the Council for International Organizations of Medical Sciences (CIOMS) [32].

### 2.4. Evaluation of the Neutralization Potency of Hyperimmunized Equine Plasma

An analytical method was applied in this study to determine the neutralization potency of antivenom prepared at Taiwanese CDC [29,33]. Briefly, 0.5 mL samples of PBS containing 4 times the minimal lethal dose (MLD) of *D. acutus* venom were incubated respectively with 0.5 mL of different dilutions of horse plasma at 37 °C for 1 h. Then, 0.2 mL of each mixture was intraperitoneally administrated into each group of ICR mice (*n* = 3). The survival rate was recorded 48 h after injection. The neutralizing potency was calculated in Tanaka units/mL by using the formula: Tanaka unit/mL = (highest plasma dilution protecting all mice) × 4 MLD × 5. Results in Tanaka units/mL indicated the amount of venom that was completely neutralized per milliliter of equine plasma.

### 2.5. Indirect-Enzyme-Linked Immunosorbent Assay (Indirect ELISA)

The protein (50 ng) was diluted with 50 µL PBS and then coated onto 96-well polystyrene microplates (Corning Inc., Corning, NY, USA), followed by incubation overnight at 4 °C. The plates were washed six times with 100 µL of PBS-T (0.1% Tween-20) and blocked with 100 µL of 1% ovalbumin in PBS for 2 h at room temperature. After six rounds of additional washing, equine plasma samples (antivenom) were diluted (1:20,000) in PBS and added to each pre-coated well for another 2 h incubation at room temperature, and excess antibodies were removed by washing 6 times with PBS-T. Afterwards, rabbit anti-horse IgG conjugated with horseradish peroxidase (HRP) (Bethyl Laboratories, Montgomery, TX, USA) was added to each well and incubated at room temperature for 1 h. After final washing six times, 50 µL of tetramethylbenzidine (TMB) substrate (Clinical Science Products Inc., Mansfield, MA, USA) was added to each well and reacted for 10 min. The reaction was terminated by adding 25 µL of 2N H_2_SO_4_ (J.T Baker, Radnor, PA, USA), and absorbance was measured using a SpectraMax M5 microplate reader (Molecular Devices, San Jose, CA, USA) with excitation and emission wavelengths of 450 and 540 nm, respectively. Each assay and sample extraction was performed in triplicate.

### 2.6. Sodium Dodecyl Sulfate-Polyacrylamide Gel Electrophoresis (SDS-PAGE)

The protein concentration of each sample was measured using a Pierce BCA protein assay kit (Thermo Fisher Scientific, Waltham, MA, USA), and ten micrograms of whole venom or 2 μg of fractionated protein analyzed by SDS-PAGE under reducing conditions. Briefly, samples were dissolved in sample buffer (125 mM Tris, 25% glycerol, 10% 2-mercaptoethanol, 4% SDS, 0.05% bromophenol blue) and heated at 95 °C for 5 min. Samples were then subjected to electrophoresis with 15% polyacrylamide gel. The protein bands resolved in SDS-PAGE gels were visualized by staining with Coomassie brilliant blue.

### 2.7. Western Blot Analysis

The venom protein was resolved by SDS-PAGE gel, transferred onto polyvinylidene difluoride (PVDF) membranes, and then probed with a 1:500 (*v*/*v*) dilution of equine plasma at 4 °C for 16 h. Antivenom-reactive proteins were detected by incubating with rabbit anti-horse IgG conjugated with HRP for 1 h, and visualized using an enhanced chemiluminescence (ECL) kit (BIONOVAS biotechnology Co., Toronto, ON, Canada). The relative photographic density was quantitated using ImageJ (National Institute of Health, Bethesda, MD, USA).

### 2.8. C18 Reverse-Phase High-Performance Liquid Chromatography (RP-HPLC) Fractionation of D. acutus Venom

The venom of *D. acutus* was separated by RP-HPLC as previously described [7]. Briefly, crude venom (100 μg protein) was dissolved in solvent A (water containing 0.1% trifluoroacetic acid (TFA)) and separated by RP-HPLC using a Supelco Discovery 300 Å C18 column (4.6 × 150 mm, 3 μm particle size) (Sigma-Aldrich, St. Louis, MI, USA). The flow rate was set to 0.7 mL/min, and the column was developed with a linear gradient of solvent A and solvent B (acetonitrile containing 0.1% TFA) as follows: isocratic (5% B) for 3 min, followed by linear gradients of 6 segments (5–10% B for 2 min, 10–16% B for 6 min, 16–28% B for 4 min, 28–41% B for 32 min, 41–52% B for 5 min, 52–80% B for 3 min) followed by an equilibrium. Peaks were detected by monitoring absorbance at 280 nm, and chromatographic fractions were collected manually. Then, collected fractions were lyophilized and stored at −20 °C.

### 2.9. In-Gel Tryptic Digestion of Protein

After staining with Coomassie brilliant blue dye, the target protein band was excised from the gel, and subjected to in-gel tryptic digestion, as previously described [34]. Briefly, gel pieces were destained three times with 40% ACN containing 25 mM ammonium bicarbonate for 15 min each, reduced with 5 mM dithiothreitol at 60 °C for 30 min, and then alkylated with 15 mM iodoacetamide at room temperature in the dark for 30 min. Proteins in the processed gel pieces were subjected to tryptic digestion with freshly prepared trypsin solution containing 20 μg/mL of trypsin (Promega, Madison, WI, USA) in 25 mM ammonium bicarbonate at 37 °C for 16 h, then extracted with 100% ACN containing 1% TFA. Finally, the extracted tryptic peptides were lyophilized and stored at −20 °C until use.

### 2.10. LC-MS/MS Analysis

The tryptic peptide from in-gel digestion was reconstituted with 5 μL of 0.1% formic acid (FA), and then analyzed using a nano-LC–LTQ-Orbitrap hybrid mass spectrometer (Thermo Fisher, San Jose, CA, USA). Briefly, the sample was loaded across a trap column (Zorbax 300SB-C18, 0.3 × 5 mm; Agilent Technologies, Wilmington, DE, USA) at a flow rate of 0.2 µL/min in HPLC buffer (0.1% FA, buffer A), and separated on a resolving 10-cm analytical C18 column (inner diameter, 75 μm) using a 15-μm tip (New Objective, Woburn, MA, USA). The peptides were fractionated using a linear gradient of 0–10% HPLC buffer B (100% ACN containing 0.1% FA) for 3 min, 10–30% buffer B for 35 min, 30–35% buffer B for 4 min, 35–50% buffer B for 1 min, 50–95% buffer B for 1 min, and 95% buffer B for 8 min, with a flow rate of 0.25 µL/min. The resolution of the Orbitrap was 30,000, and the ion signal of (Si(CH_3_)_2_O)_6_H^+^ at 445.120025 (*m*/*z*) was used as a lock mass for internal calibration. A procedure was applied alternating between one MS scan and 10 MS/MS scans for the 10 most abundant precursor ions. The *m*/*z* values selected for MS/MS were dynamically excluded for 180 s. The *m/z* value of the MS scan range was 400 to 2000 Da. For MS/MS scans, more than 1 × 10^4^ ions were accumulated in the ion trap to generate the MS/MS spectra. MS and MS/MS spectra were both acquired using a single scan with maximum fill times of 1000 and 100 ms for MS and MS/MS analysis, respectively. For database searching, raw MS data files were analyzed by Proteome Discoverer software (version 2.3.0.523; Thermo Fisher, San Jose, CA, USA), and were searched against lobe-finned fish and tetrapod clade taxonomy in the Swiss-Prot database, using MASCOT (Matrix Science Inc., Boston, MA, USA). The parameter of enzyme specificity was set to “trypsin” and one missed cleavage was permitted. Carbamidomethylation of cysteine was set as a static modification and oxidation of methionine, acetyl (protein N-term), and Gln- > pyro-Glu (N-term Q) was set for dynamic modification. The tolerance of MS was 10 ppm, and that of MS/MS was 0.6 Da. The criteria of minimum number of peptides per identified protein was 2, containing at least one unique peptide.

### 2.11. Statistical Analysis

Statistical analysis was performed using two-sample *t* testing. All statistical analyses were performed using Graphpad Prism 5 software (La Jolla, CA, USA). Differences were considered statistically significant at *p*-value ≤ 0.05.

## 3. Results

### 3.1. Neutralization Potency of Equine Plasma against Deinagkistrodon acutus Venom

Two individual horses (No. 341 and 410) were concurrently immunized with *D. acutus* venom for eight cycles before analysis of neutralization potency in vivo. In the rodent model testing, the two hyperimmunized equine plasma samples showed obvious differences in neutralizing the lethality of *D. acutus* venom, at 100 and 20 Tanaka units/mL, respectively (Table 1). Notably, the neutralization potency of No. 341 was significantly higher than that of No. 410 under the same immunization conditions. To clarify the mechanisms underlying the differences between the two hyperimmunized horses, the respective plasma samples (categorized as high and low potency) were used as models for further experiments.

### 3.2. Comparison of the Efficacy of High-Potency and Low-Potency Horse Plasma against Crude Venom of D. acutus

**Figure 1:**
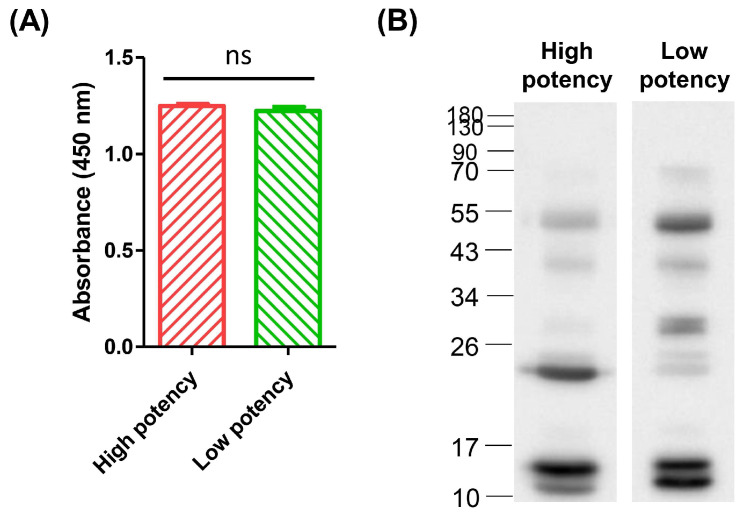
Immunoprofiles of high-potency and low-potency horse-plasma samples against whole *D. acutus* venom. (**A**) The venom of hundred-pace pit vipers was initially coated on the microplate and indirect ELISA was performed using high- and low-potency horse plasma as primary antibodies. Each bar represents means ± SD of triplicates (ns: not significant). (**B**) Western blot analysis of the proteins involved in immune reactions of both horse-plasma samples against whole venom. The densitometry intensity ratio of each band is shown in Supplementary Materials Table S1.

Indirect ELISA was conducted to determine the differences in antibody titers between high-potency and low-potency horse plasma against whole *D. acutus* venom. Venom proteins were coated onto a 96-well microplate and horse plasma served as the primary antibody for probing. The absorbance signal did not indicate significant differences between high- and low-potency plasma samples (Figure 1A), indicating that the number of antibodies against whole *D. acutus* venom in low-potency plasma was comparable to that in high-potency plasma.

Western blot analysis was then performed to determine the target proteins recognized by the two equine plasma samples. Three main protein bands were recognized by low-potency plasma, located at 55 kDa and ~10–15 kDa (Figure 1B). Immunoprofiling revealed higher affinity of high-potency equine plasma for protein bands located at 17–26 kDa and ~15 kDa, and the ratios of densitometric intensity for high-potency and low-potency samples were 425% and 150%, respectively (Supplementary Materials Table S1). Moreover, three different individuals immunized in the next schedule under identical conditions displayed low neutralization potency and were further analyzed by Western blotting. All of these consistently showed relatively lower immunoreactions against the protein band located at 17–26 kDa (Supplementary Materials Figure S1). In view of the notable finding that the protein band at ~17–26 kDa was specifically immunocaptured by high-potency equine plasma, we hypothesized that this recognition response may serve as a key contributory factor in the neutralization ability of equine plasma from different hyperimmunized horses.

### 3.3. Fractionation of Venom Protein Components from D. acutus

Based on the above findings, we further explored the theory that antibodies bound to the 17–26 kDa protein may underlie the major differences in neutralization activity between high- and low-potency horse plasma. To investigate the proteins in this range of molecular mass, whole *D. acutus* venom was fractionated using RP-HPLC following an established protocol [7].

**Figure 2:**
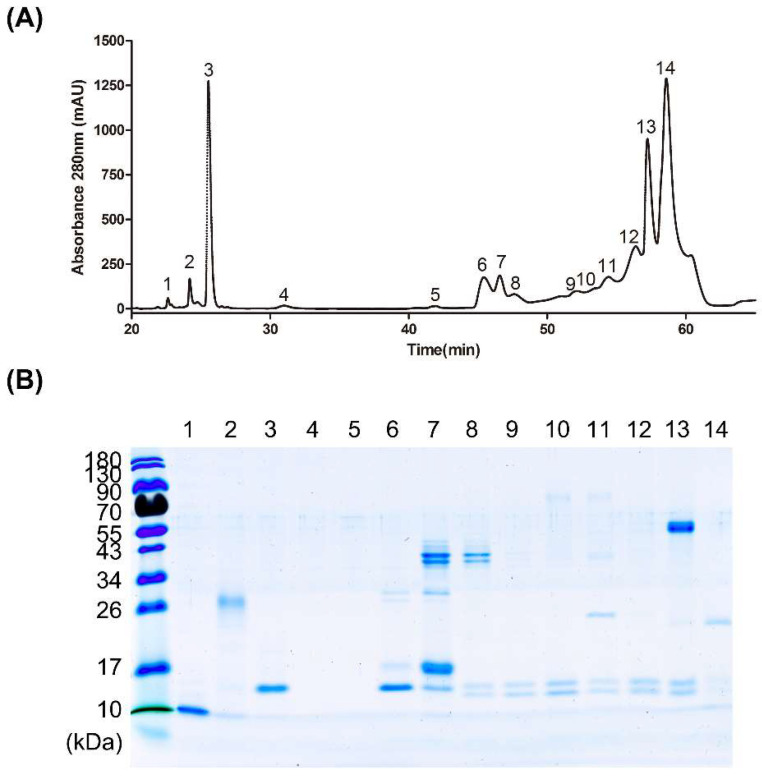
Protein profile of HPLC-separated *D. acutus* venom. (**A**) Crude venom was fractionated via RP-HPLC and 14 fractions were collected from the peak signals at an absorbance of 280 nm. (**B**) Fractionated proteins from *D. acutus* venom were analyzed via SDS-PAGE and visualized with Coomassie blue staining. The intensity of bands indicates relative abundance of proteins.

After separation by liquid chromatography, fractionated proteins were identified via spectrophotometry with absorbance at 280 nm. Crude *D. acutus* venom was resolved into 14 protein fractions via RP-HPLC, with the majority eluting between 40 and 60 min (Figure 2A). Each fraction was subjected to SDS-PAGE followed by Coomassie blue staining (Figure 2B). Most proteins were distributed at three major molecular weights, specifically, 55 kDa, 17–26 kDa and 10–17 kDa. The 55 kDa protein was visible in fraction 13 while fraction 14 contained distinct protein bands at 17–26 kDa. Distinct bands at 10–17 kDa were detected in fractions 6 to 13. More than one protein band was observed for the majority of fractions. Fractions 4 and 5 contained no protein bands and were therefore excluded from further analysis.

### 3.4. Affinity of High-Potency and Low-Potency Horse Plasma for Protein Fractions from D. acutus Venom

Indirect ELISA was conducted to examine the differences in the affinity of high- and low-potency equine plasma samples for the 14 protein fractions. In lieu of crude venom, HPLC-separated venom proteins were coated onto a microplate and reacted with both plasma samples.

**Figure 3:**
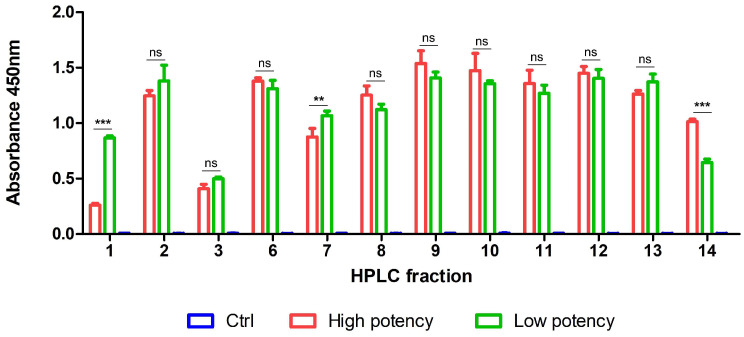
Immunorecognition of fractionated venom proteins by high-potency and low-potency horse plasma samples. A microplate was coated with 14 protein fractions of HPLC-separated *D. acutus* venom and incubated with both plasma samples, respectively, for indirect ELISA analysis. Each bar represents mean ± SD of triplicates (** *p* ≤ 0.01; *** *p* ≤ 0.001; ns: not significant).

For the majority of fractions, no significant differences were observed between absorbance of high-potency and low-potency plasma (Figure 3). Notably, however, high-potency equine plasma showed higher affinity than low-potency plasma for fraction 14. This observation supports the hypothesis that high-potency plasma has better ability to neutralize the specific protein toxin of the hundred-pace pit viper that is mainly associated with poisoning.

While a clear difference was observed between the absorbance effects of high-potency and low-potency plasma on fractions 1 and 7, the higher detection signal for low-potency plasma was inconsistent with our hypothesis. Based on the results, we suggest that components of fraction 14 play an important role in conferring the high potency of specific equine plasma against venom proteins.

### 3.5. Differences in Antibody Titers against D. acutus Venom between High- and Low-Potency Horse Plasma

**Figure 4:**
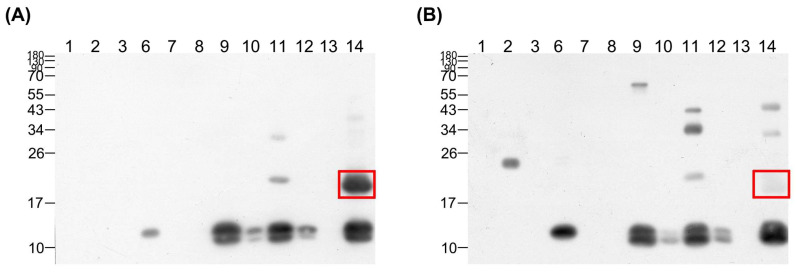
Western blot analysis of high-potency and low-potency horse plasma against HPLC-fractionated venom from hundred-pace pit vipers. HPLC-separated venom proteins were transferred to the PVDF membrane following SDS-PAGE and were probed with (**A**) high-potency and (**B**) low-potency plasma. The significant difference between the immunoprofiles of the two plasma samples is highlighted with a red box.

To further establish the differences in venom proteins recognized by the two plasma samples, Western blotting was performed, and individual protein fractions were probed with each plasma sample. In our immunoblot experiments, both plasma samples recognized proteins at 10–17 kDa from fraction 6 and fractions 9 to 14 (Figure 4). The protein band at 17–26 kDa in fraction 14 was specifically recognized by the high-potency equine plasma, while no clear signal was evident in this area for the low-potency plasma, suggesting that the antibody recognizing the ~20 kDa protein in fraction 14 may serve as the decisive factor in the high antivenom efficacy of horse plasma.

**Figure 5:**
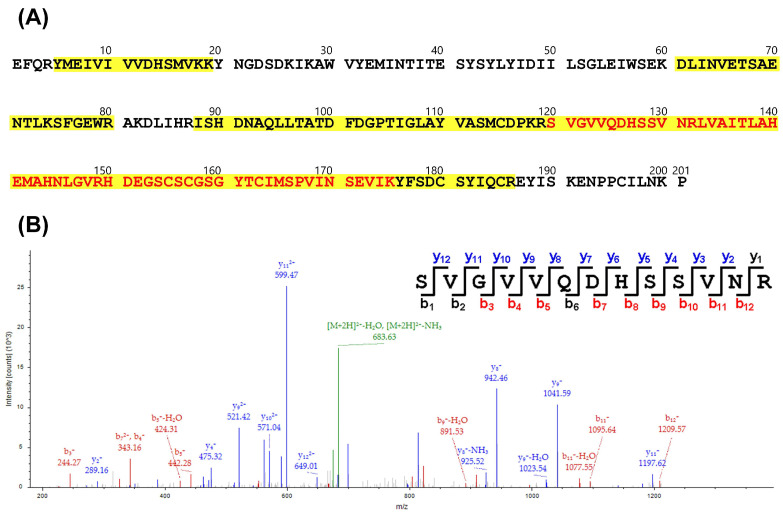
Identification of acutolysin A by LC–MS/MS analysis. (**A**) The positions of identified peptides were indicated in the full-length sequence of acutolysin A, and the unique peptides are denoted in red. (**B**) The MS/MS spectrum presents the identification of unique peptide SVGVVQDHSSVNR.

To characterize the 20 kDa protein of fraction 14, LC-MS/MS was performed. The target protein band was separated via SDS-PAGE (Figure 2B), prepared using in-gel digestion, and subsequently analyzed with mass spectrometry. Comparison of the spectrometry patterns with the database led to the identification of this protein as acutolysin A, a critical toxin of *D. acutus* venom. The results indicated that seven tryptic peptides including three unique peptides were identified for acutolysin A (Figure 5A), covering 67% of the full-length sequence, and a representative MS/MS spectrum of one unique peptide is shown (Figure 5B and Supplementary Materials Figure S2).

## 4. Discussion

The immunorecognition profiles of high-potency and low-potency equine plasma samples against the venom of the five-pace snake were investigated in order to examine the differences between the target venom proteins of the two groups. Our results showed that the most significant difference between the immunoprofiles of the high- and low-potency horse plasma was observed in the antibody titer against acutolysin A.

*D. acutus* venom consists of four dominant protein families, specifically, snake venom metalloproteinase (SVMP), snake venom serine protease (SVSP), phospholipase A2 (PLA2), and snake venom C-type lectin (CLEC) [7,35]. Acutolysin A, also known as hemorrhagin I (HI), is a member of the SVMP family with a molecular weight of 22 kDa and can be isolated from *D. acutus* venom [36]. Proteins belonging to SVMP contain at least one zinc-binding domain [37,38] and can cause degradation of the extracellular matrix, disruption of cellular interactions, and breakdown of capillaries and blood vessels, leading to local and systemic hemorrhage [39,40]. Acutolysin A is the most hemorrhagic component in the venom of the hundred-pace pit viper [41].

In view of the major difference between high-potency and low-potency plasma groups revealed in the antibody titer against acutolysin A, we propose that acutolysin A may serve as a key determinant of the neutralization potency of hyperimmunized horse plasma. Previous reports have documented that patients bitten by *D. acutus* usually suffer from continuous oozing of interstitial fluid from the fang marks and painful swelling extending to body parts near the envenomed region [42].If treatment with specific antivenom is delayed or not administered, exacerbated conditions such as tissue ecchymosis and gross hematuria may occur due to the tendency for excessive bleeding conferred by *D. acutus* venom. In some cases, patients present multiple hemorrhagic bullae scattering along the limbs and bleeding in organs distant from the site of envenomation, which can increase the pressure within muscles to a dangerous level and lead to compartment syndrome. Without proper medical care, limb-threatening complications including wound infections and large areas of muscle necrosis can additionally occur days after snakebite [43,44,45]. Systematic hemorrhage is a life-threatening symptom of *D. acutus* envenomation.

Acutolysin A is one of the most toxic hemorrhagic proteins in *D. acutus* venom [41]. Lack of antibodies in horse plasma against this venom protein could influence its inhibitory potency toward hemorrhagic activity of *D. acutus* venom and consequently decrease the neutralization potency of anti-*D. acutus* antivenom. Our theory that the ability of antivenom to prevent lethal symptoms of *D. acutus* envenomation may be highly related to the variable affinity of equine plasma for acutolysin A is supported by this perspective. Further investigations on the relative toxicity and lethality of acutolysin A are essential for the development of strategies to improve the neutralization potency of *D. acutus* antivenom.

The lack of affinity in antivenom for major toxin proteins is a critical issue worldwide affecting therapy for snakebites, and several novel strategies for antivenom production and snakebite treatment have been developed in an attempt to resolve this problem [20].

Booster injections of recombinant venom components instead of crude venom have been examined as a potential method to improve the clinical efficacy of antivenom. Utilization of major toxins from snake venom for immunization has been reported to stimulate pathology-specific antibodies to a greater extent than whole venom and to improve overall antivenom efficacy [46]. This result supports the concept of additional hyper-immunization of optimal venom components, which are highly toxic but weakly immunogenic, to improve neutralizing efficacy against specific venom proteins. Accordingly, boosting of major toxins to stimulate additional antivenom activity may be a feasible solution to address the lack of neutralization potency in antivenom.

Furthermore, supplementation of traditional antivenom with monoclonal antibodies targeting highly toxic proteins may promote neutralizing potency to a standard that meets harvesting criteria. Recently, the neutralization abilities of oligoclonal human IgG antibodies and single-chain variable fragments (scFv) were demonstrated to be equivalent to or greater than those of animal-derived polyclonal antibodies [47,48]. In this respect, addition of the desired therapeutic antibodies that may be lacking in hyper-immunized horse plasma may improve therapeutic efficacy of the antivenom that is produced.

Finally, combination of antivenom and small-molecule drugs that inhibit major toxins could potentially circumvent the long-existing problem of insufficient dose efficacy [49,50]. Taking metalloproteinase (MMP) inhibitors as an example, marimastat capable of inhibiting metalloproteinase activity effectively decreased the toxicity of SVMP activity in the venom of the saw-scaled viper, *Echis ocellatus*, and increased survival in the venom-injected mouse model (up to 80%) [51]. Thus, combination of traditional antivenom with repurposed MMP inhibitor drugs for envenomation treatment may present a feasible strategy to enhance the efficacy of horse-derived antivenom in preventing SVMP toxicity.

Although accurate evaluation of the toxicity of acutolysin A in *D. acutus* venom remains to be completed, this investigation suggest that acutolysin A may serve as an important factor related to the neutralization potency of *D. acutus* antivenom. To further improve the efficacy of anti-*D. acutus* antivenom titer against this component, the three strategies discussed above are feasible options, i.e., additional boosting of acutolysin A, or supplementation with anti-acutolysin A antibodies or SVMP inhibitors.

## 5. Conclusions

In summary, our study focused on the immunoreactions of anti-*D. acutus* equine plasma samples with high and low neutralization potencies towards individual components of venom, and revealed that antibodies against acutolysin A were accountable for the differences in potency of antivenom from different horses hyperimmunized with *D. acutus* venom. Our findings offer novel insights into the immune responses of hyperimmunized horses to *D. acutus* venom and provide a basis for designing innovative strategies for anti-*D. acutus* antivenom production. These clues may advance antivenom manufacture and further improve the efficacy of treatment against *D. acutus* envenoming in the future.

## Figures and Tables

**Table 1:** Evaluation of neutralization potency of hyperimmunized equine plasma.

No.	Group	Administrated Venom ^a^	Plasma	Dilution Buffer ^b^	Survival
		(mL)	(mL)	(mL)	(*n* = 3)
341	A	0.5	0.5	0	3/3
341	B	0.5	0.25	0.25	3/3
341	C	0.5	0.166	0.334	3/3
341	D	0.5	0.125	0.375	3/3
341	E	0.5	0.1	0.4	3/3
341	F	0.5	0.083	0.417	1/3
410	A	0.5	0.5	0	3/3
410	B	0.5	0.25	0.25	2/3
410	C	0.5	0.166	0.334	0/3
410	D	0.5	0.125	0.375	0/3
410	E	0.5	0.1	0.4	0/3
410	F	0.5	0.083	0.417	0/3

^a^ The concentration of administrated venom was 4 MLD/mL, and the MLD of *D. acutus* venom was 60.8 μg. ^b^ PBS served as the diluent.

## Data Availability

Not applicable.

## Supplementary Materials

The following supporting information can be downloaded at: https://www.mdpi.com/article/10.3390/tropicalmed8010051/s1, Figure S1: The immunoprofiling of low-potency equine plasma from three individuals against whole *D. acutus* venom; Figure S2: MS/MS spectrum of two unique peptides of acutolysin A; Table S1: The ratio of blotting bands between high-potency and low-potency equine plasma under densitometry analysis.
