# Supplementary figures and images for: Immunoprofiling of Equine Plasma against Deinagkistrodon acutus in Taiwan: Key to Understanding Differential Neutralization Potency in Immunized Horses
Source: Trop Med Infect Dis. 2023 Jan 9;8(1):51. doi: 10.3390/tropicalmed8010051 (PMC9866385; doi:10.3390/tropicalmed8010051)

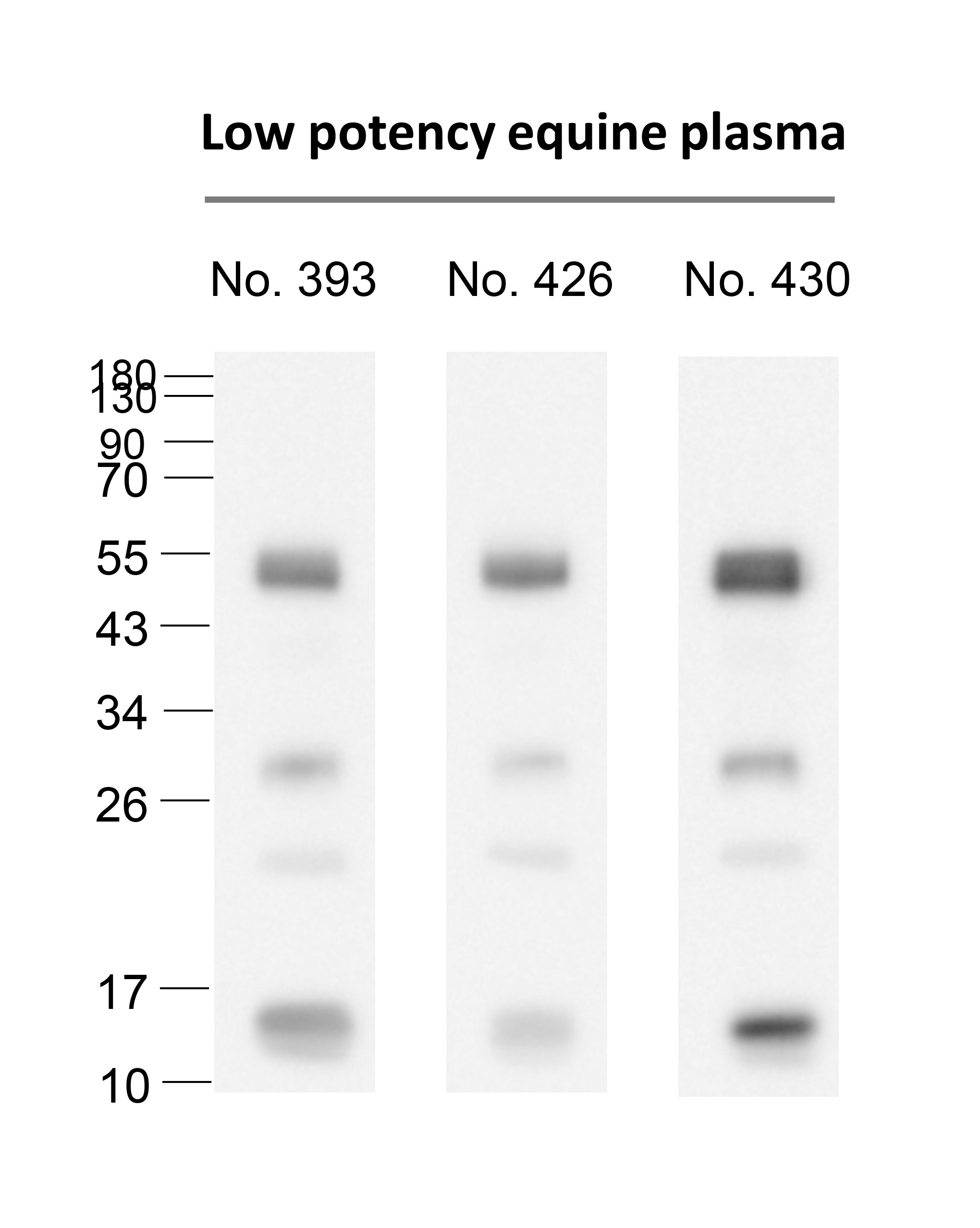

Supplement: Supplementary file 1 [file tropicalmed-08-00051-s001.zip › tropicalmed-2071230-supplementary/Supplemental File/Fig. S1.tif]

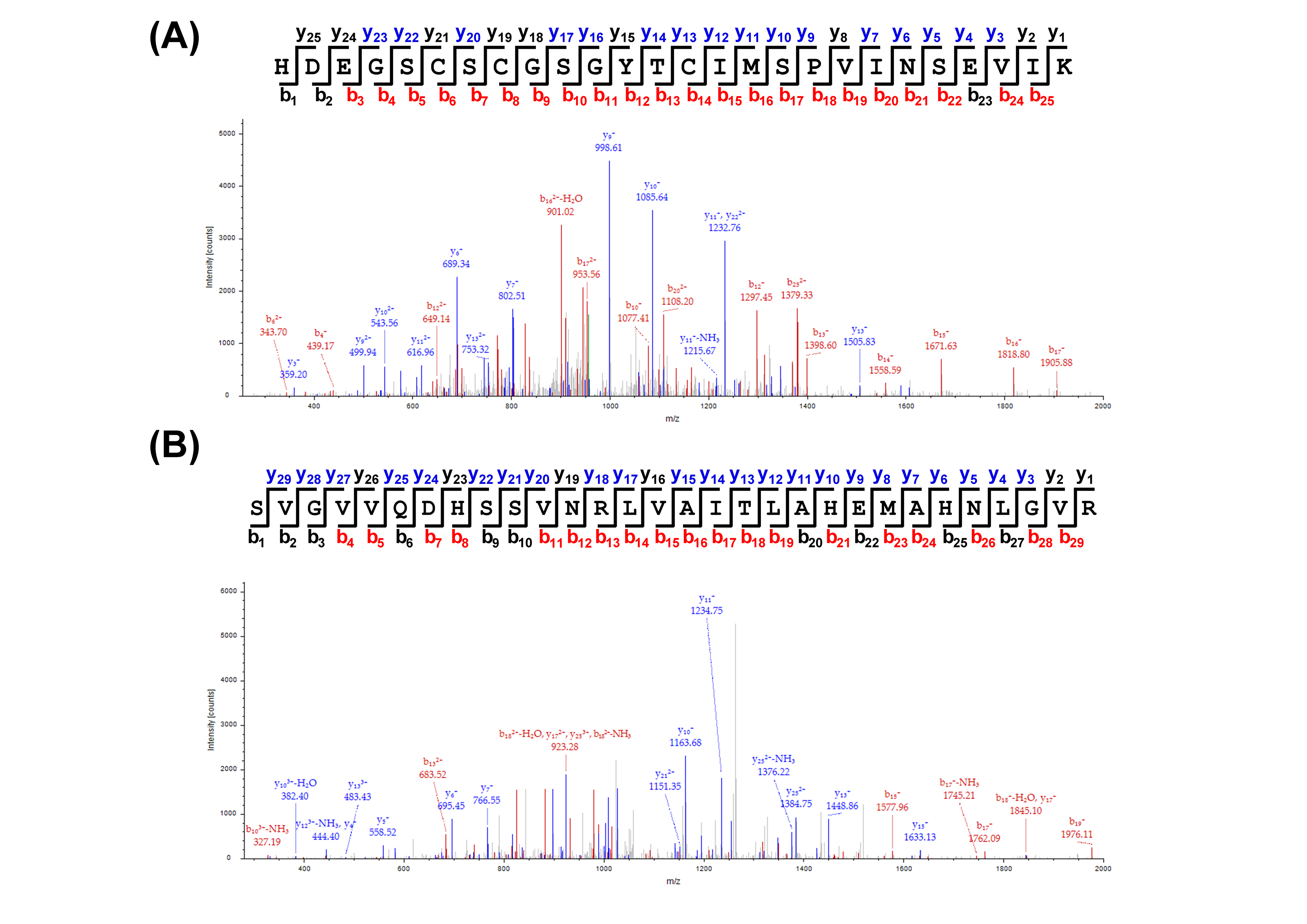

Supplement: Supplementary file 1 [file tropicalmed-08-00051-s001.zip › tropicalmed-2071230-supplementary/Supplemental File/Fig. S2.tif]
